# Municipal wealth and tooth extractions in the Unified Health System: limits to changing the healthcare model

**DOI:** 10.11606/s1518-8787-2026060007600

**Published:** 2026-09-02

**Authors:** Cristiane Maria da Costa Silva, Juliane Franco Martins, Gustavo Fernando Silva, Walbert de Andrade Vieira, Carlos José Soares, Luiz Renato Paranhos

**Affiliations:** I Universidade Federal de Uberlândia Faculdade de Odontologia Programa de Pós-Graduação Uberlândia MG Brasil Universidade Federal de Uberlândia. Faculdade de Odontologia. Programa de Pós-Graduação. Uberlândia, MG, Brasil; II Universidade Federal de Uberlândia Faculdade de Odontologia Uberlândia MG Brasil Universidade Federal de Uberlândia. Faculdade de Odontologia. Uberlândia, MG, Brasil; III Centro Universitário das Faculdades Associadas de Ensino Faculdade de Odontologia São João da Boa Vista SP Brasil Centro Universitário das Faculdades Associadas de Ensino. Faculdade de Odontologia. São João da Boa Vista, SP, Brasil; IV Universidade Federal de Uberlândia Faculdade de Odontologia Departamento de Dentística e Materiais Dentários Uberlândia MG Brasil Universidade Federal de Uberlândia. Faculdade de Odontologia. Departamento de Dentística e Materiais Dentários. Uberlândia, MG, Brasil; V Universidade Federal de Uberlândia Faculdade de Odontologia Departamento de Ortodontia Uberlândia MG Brasil Universidade Federal de Uberlândia. Faculdade de Odontologia. Departamento de Ortodontia. Uberlândia, MG, Brasil

**Keywords:** Tooth Extraction, Unified Health System, Primary Health Care, Time Factors, Development Indicators

## Abstract

**OBJECTIVE:**

To analyze the time trend of dental extractions performed in primary health care and their association with human development indicators and oral health coverage in municipalities at the extremes of wealth distribution.

**METHODS:**

An ecological study using secondary data from August 2019 to July 2024, with Brazilian municipalities as the units of analysis. A total of 200 municipalities were selected, comprising the 100 with the lowest and the 100 with the highest gross domestic product *per capita*. The number of tooth extractions was obtained from national health information systems, and rates were calculated per 1,000 inhabitants. The temporal trend was estimated using generalized least squares regression with an autoregressive structure, and the annual percentage change was calculated. Comparisons between the groups were performed using a mixed-effects model with a negative binomial distribution, adjusted for indicators of oral health coverage, socioeconomic status, and demographics. A significance level of 5% was used.

**RESULTS:**

A total of 355,218 tooth extractions were recorded during the study period. The rates showed stationary trends in both groups, although municipalities with higher *per capita* gross domestic product exhibited a borderline trend towards an increase (p = 0.06). The Municipal Human Development Index was inversely associated with the outcome, while population coverage of primary patternsoral health care was positively associated with it. *Per capita* gross domestic product was not significantly associated with tooth extraction rates in the adjusted model.

**CONCLUSION:**

The stability of tooth extraction rates throughout the time series, regardless of municipal wealth, suggests that factors related to human development and the organization of services may be more relevant for understanding the distribution of this procedure within the Unified Health System.

## INTRODUCTION

Tooth extractions are among the most commonly performed dental procedures in the Unified Health System (SUS) and constitute a relevant indicator of access patterns to oral health care in Primary Health Care (PHC)^1,2^. Although they are necessary interventions, their high frequency may reflect delayed access, low clinical effectiveness, and persistent social inequalities in dental care^3,4^. In Brazil, these inequalities manifest in varied ways across municipalities with different levels of human development, influencing both the demand for and the capacity to provide services^5,6^.

Regions with lower Human Development Index (HDI) scores tend to have poorer access indicators^7^, a higher burden of oral diseases^8^, and delayed diagnosis of oral cancer^9^. However, the tooth extraction patterns remain underexplored from the perspective of inequalities among municipalities. Among the causes of tooth loss are dental caries^10,11^, followed by periodontal diseases and trauma^12,13^, which represent global public health problems, especially when considering that billions of people lack access to basic oral health care^14^. When conservative treatment is not feasible, tooth extraction becomes the most financially accessible option for pain relief^15,16^. In this context, persistently high rates of tooth extraction may exacerbate inequalities in oral health.

The loss of teeth can significantly impact an individual’s quality of life, since, in addition to the aesthetic implications that affect social interactions, there is also functional deterioration that impairs speech and masticatory function^16^, even though prosthetic rehabilitation is an option. In contexts with poorer social and economic indicators, access to oral health services^17^ and, consequently, to prosthetic rehabilitation^18^ is often more limited, which exacerbates the social and functional effects of tooth loss.

Despite the importance of this topic, it remains unclear whether differences in municipal wealth can influence the temporal patterns of tooth extractions in PHC. This knowledge is essential for identifying structural inequalities, guiding regionalization strategies, and strengthening evidence-based oral health policies. Thus, the objective of this study was to analyze the temporal trend of tooth extractions performed in the SUS primary care system and their association with municipal indicators of wealth and human development.

## METHODS

### Study Type

This ecological study used publicly available secondary data covering the period from August 2019 to July 2024. As it does not involve individual identification, the study is exempt from review by a Research Ethics Committee, in accordance with National Health Council Resolution No. 510/2016. The reporting of this study followed the STROBE (Strengthening the Reporting of Observational Studies in Epidemiology)^19^ guidelines and its extension RECORD (REporting of studies Conducted using Observational Routinely-collected health Data)^20^, which are specific to research based on routinely collected secondary data.

### Participants

The study population consisted of Brazilian municipalities, considered as units of analysis, classified according to municipal *per capita* gross domestic product (GDP). Municipalities were eligible if they had valid tooth extraction records in the SUS between August 2019 and July 2024.

For analytical purposes, the municipalities were stratified into two extreme groups based on municipal wealth, defined according to the distribution of *per capita* GDP: one group comprising the 100 municipalities with the lowest *per capita* GDP and the other comprising the 100 municipalities with the highest *per capita* GDP. This selection was analytical and exploratory in nature, with the aim of comparing markedly distinct economic contexts and determining whether marked differences in municipal wealth were associated with tooth extraction rates in primary care. The study included all municipalities belonging to one of these two strata that had at least one record of tooth extraction during the evaluation period. Municipalities with no records of the procedure were excluded from the analysis.

### Sample Size and Selection

The sample size was determined to ensure enough municipalities for comparison between socioeconomic strata. The number of municipalities classified as having high social vulnerability by the Institute of Applied Economic Research (IPEA)^a^, was used as a reference for the sample size calculation; a 95% confidence interval and a 10% sampling error, plus a 5% safety margin to account for possible data inconsistencies.

Based on these parameters, 100 municipalities were selected for each group, corresponding to the extremes of the distribution of municipal GDP *per capita*. For the selection, all Brazilian municipalities were ranked in ascending order of GDP *per capita*, based on data from the Brazilian Institute of Geography and Statistics (IBGE)^b^ and the 100 municipalities with the lowest and the 100 with the highest GDP *per capita* were selected.

### Variables

The dependent variable was the number of extractions of primary and permanent teeth performed during the analyzed period, identified by the codes 04.14.02.012-0 (primary) and 04.14.02.013-8 (permanent).

The independent variables included: GDP *per capita*, Municipal Human Development Index (MHDI), Gini Index, population coverage by primary oral health care, total population, and population density of the selected municipalities.

### Data Collection

Data on tooth extractions performed by Brazilian municipalities were obtained from the Departamento de Informática do Sistema Único de Saúde (Datasus – Information Technology Department of the Unified Health System) through two systems: the Sistema de Informações Ambulatoriais^c^ (SIA/SUS – Outpatient Information System) and the Sistema de Informação em Saúde para a Atenção Básica^d^ (Sisab – Health Information System for Primary Care), both of which are publicly accessible.

The concurrent use of these systems is justified by changes in the organization of outpatient PHC service records in Brazil throughout the analyzed period, especially following the consolidation of the e-SUS primary care system. Thus, the integration of information was necessary to ensure the completeness and temporal continuity of the data, minimizing information losses resulting from the transition between systems.

Tooth extractions were identified using the official codes from the Sistema de Gerenciamento da Tabela de Procedimentos, Medicamentos e Ooteses, Próteses e Materiais do SUS (SIGTAP – SUS Management System for the Table of Procedures, Medications, and Orthoses, Prostheses, and Special Materials), including only records corresponding to extractions of permanent teeth (04.14.02.013-8) and deciduous teeth (04.14.02.012-0), performed between August 2019 and July 2024. Tooth extraction procedures involving supernumerary teeth were excluded.

**Table:** Sociodemographic characteristics and tooth extraction rates of the municipalities included in the study, according to municipal wealth group.

	Higher GDP m (SD)	Lowest GDP m (SD)
*Per capita* GDP	128,321.9 (147.4)	7,180.7 (150.7)
Population	39,030.06 (74,539.0)	16,214.78 (8,633.5)
Primary care oral health coverage	76.5 (32.4)	91.7 (18.4)
Human Development Index	0.699 (0.06)	0.557 (0.03)
Gini Index	0.40 (0.04)	0.37 (0.03)
Population density	168.96 (560.79)	26.74 (24.79)
Tooth loss rate^a^	11.2 (14.7)	19.1 (18.4)
Total extractions	271.9 (771.7)	288.3 (303.6)

SD: standard deviation; GDP: gross domestic product.

^a^ Rate per 1,000 inhabitants.

Population coverage for primary oral health care was obtained from the Ministry of Health’s Sistema e-Gestor (e-Manager system). Municipal socioeconomic data were obtained from the IBGE, including total population and population density (2022 Census) and GDP *per capita* (2021). The MHDI was obtained from the IBGE portal, and the Gini Index through Datasus. The data were tabulated in Microsoft Excel spreadsheets and organized by year, region, state, and municipality.

### Data Processing

Data processing included checking for duplicates, identified by records with identical information keys (municipality, reporting period, procedure code, and quantity) in the information systems, retaining only one valid record per occurrence. A temporal and numerical consistency analysis was also conducted, excluding values that were clearly incompatible with historical patterns of municipal production, thereby reducing potential biases resulting from recording errors. After these steps, the data were aggregated by municipality and by year, forming the final analytical dataset.

### Data Analysis

To assess the temporal trend in tooth extraction rates over the five-year period, rates adjusted per 1,000 inhabitants were calculated for each municipality. The annual trend was estimated separately for groups of municipalities with lower and higher GDP *per capita* using generalized least squares regression with a first-order autoregressive structure [GLS AR (1)]. Based on the coefficient of the time variable, the annual percentage change (APC) was calculated, with p > 0.05 considered a stationary trend, positive values indicating an increase, and negative values indicating a decrease.

To compare tooth extraction rates between the groups over the period, a generalized mixed-effects model (GLMM) with a negative binomial distribution (nbinom2) was fitted. The outcome was the number of procedures, adjusted by the logarithm of the municipal population as an offset (per 1,000 inhabitants). Fixed effects were included for socioeconomic group, year, MHDI, Gini index, and population density, as well as primary care oral health coverage, and the interaction between group and year. The municipality was included as a random effect to correct for intramunicipal correlation. Results were presented as rate ratios (RR), with 95% confidence intervals and p-values. Analyses were performed using R software (version 4.5.1).

## RESULTS

The analyses included 200 Brazilian municipalities (100 with the lowest and 100 with the highest GDP *per capita*), selected according to the criteria described above. From August 2019 to July 2024, the selected municipalities performed 355,218 tooth extractions through the SUS. Of this total, 51.8% were performed by municipalities classified as the lowest GDP group. Table summarizes the characteristics of the analyzed municipalities according to municipal wealth groups. In general, municipalities with lower GDP *per capita* had greater oral health coverage and a higher average rate of tooth extractions per 1,000 inhabitants, while municipalities with higher GDP *per capita* had higher average values for population density and the MHDI.

**Figure 1:**
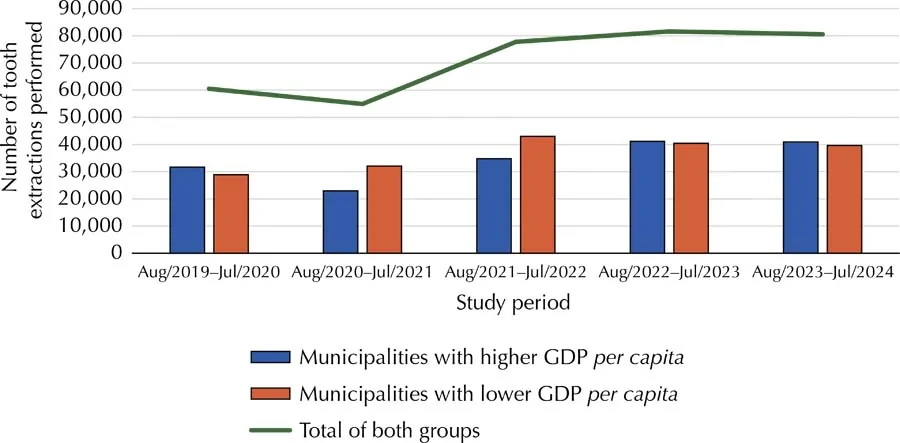
Number of tooth extractions performed in primary health care in municipalities at the extremes of GDP *per capita*. Brazil, August 2019 to July 2024.

Note: Data extracted in an integrated manner from the *Sistema de Informações Ambulatoriais* (SIA/SUS – Outpatient Information System) and the *Sistema de Informação em Saúde para a Atenção Básica* (Sisab – Health Information System for Primary Care) of the Ministry of Health. “Total for both groups” represents the sum of the procedures performed in the 200 municipalities analyzed.

Figure 1 shows the temporal evolution of the absolute number of tooth extractions performed in the municipalities included in the study, according to municipal wealth groups, as well as the aggregate total of procedures. In the first period evaluated (August 2019 to July 2020), municipalities with higher GDP *per capita* had a higher absolute number of tooth extractions compared to municipalities with lower GDP *per capita*. Between August 2020 and July 2022, a higher absolute number of procedures was observed in municipalities with lower GDP *per capita*. In the two subsequent periods, the figures became more similar across the groups. Considering all the municipalities analyzed, the total number of tooth extractions increased between August 2020 and July 2023, followed by a slight decrease in the period from August 2023 to July 2024, while remaining, however, above the values observed at the beginning of the time series.

**Figure 2:**
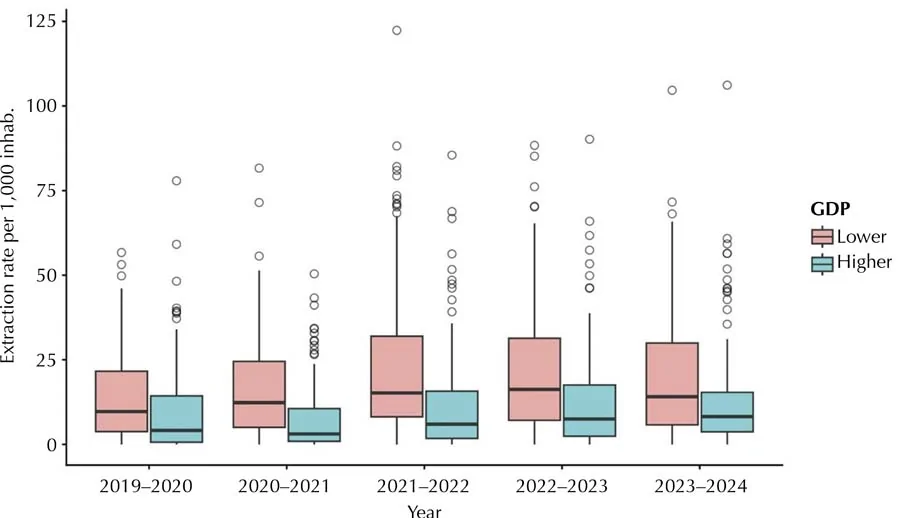
Annual rate of tooth extractions in the SUS in Brazilian municipalities, by municipal GDP. Brazil, August 2019 to July 2024.

Figure 2 shows the annual distribution of tooth extraction rates per 1,000 inhabitants by municipal wealth group. In all periods evaluated, municipalities with lower GDP *per capita* had higher medians than those with higher GDP *per capita*. Despite this, wide intragroup variability was observed throughout the entire series, with greater dispersion in some periods and the occurrence of extreme values in both strata.

GDP: gross domestic product.

**Figure 3:**
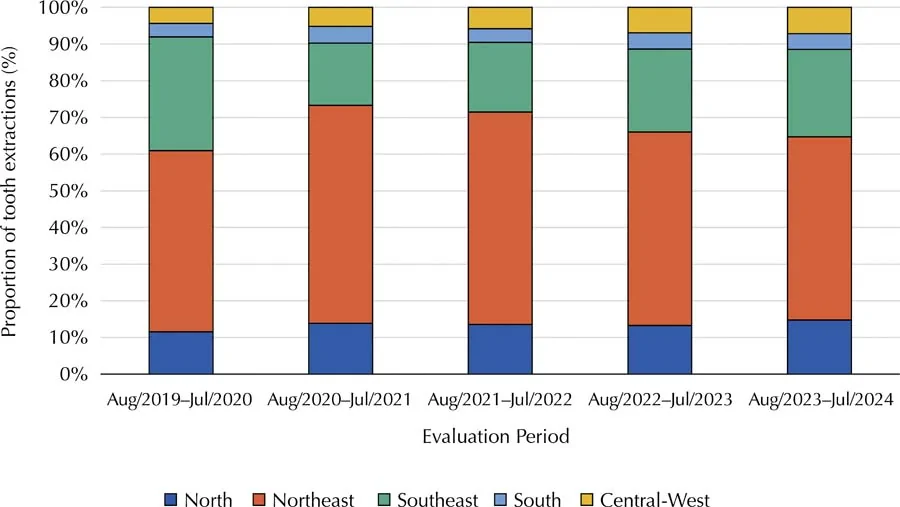
Annual proportion of tooth extractions performed by Brazilian macroregions within the SUS. Brazil, August 2019 to July 2024.

Note: Integrated data from the *Sistema de Informações Ambulatoriais* (SIA/SUS – Outpatient Information System) and the *Sistema de Informação em Saúde para a Atenção Básica* (Sisab – Health Information System for Primary Care) of the Ministry of Health. The values represent the percentage share of each macroregion in the total number of procedures performed by the municipalities in the sample.

Figure 3 shows the proportional distribution of tooth extractions by macroregion over the analyzed period. In every year, the largest proportion of procedures was concentrated in the Northeast region, followed by the Southeast and North regions. The South and Central-West regions had the lowest relative shares. This pattern remained relatively stable throughout the series, with slight variations between the evaluated periods.

**Figure 4:**
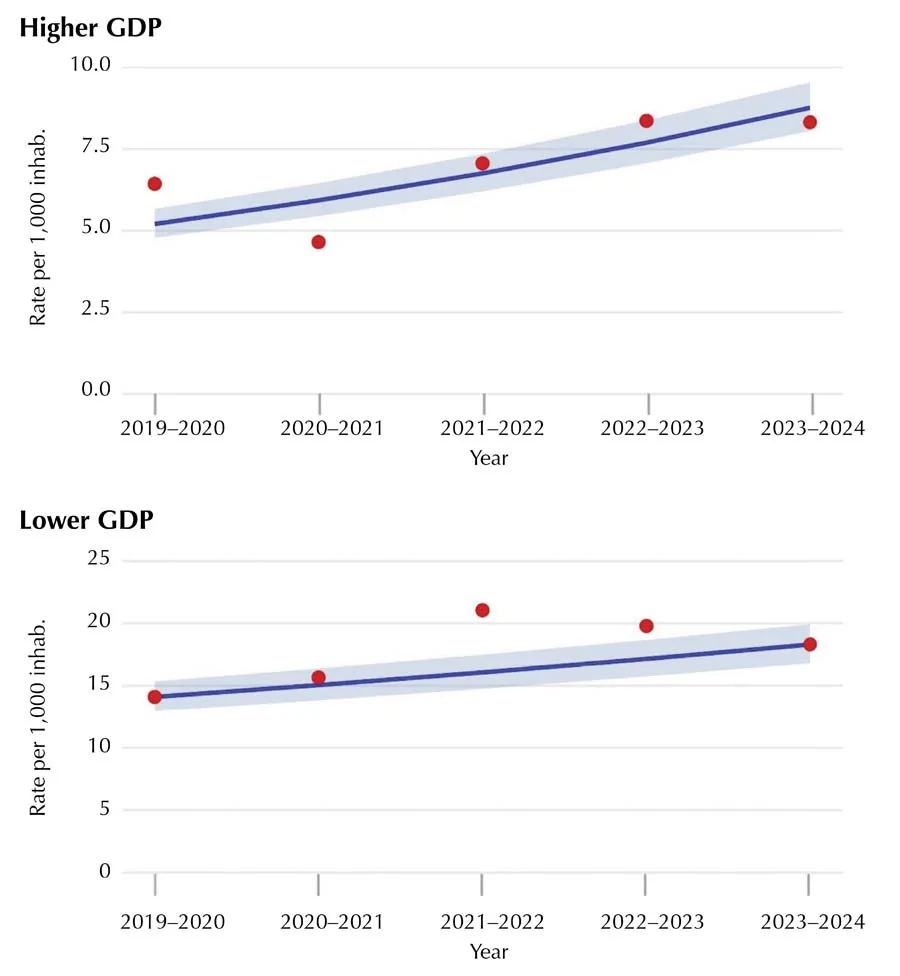
Annual percentage change in the rate of tooth extractions per 1,000 inhabitants in municipalities with the highest GDP (top) and the lowest GDP (bottom). Brazil, August 2019 to July 2024.

In the temporal trend analysis, tooth extraction rates per 1,000 inhabitants showed a stationary trend in municipalities with lower GDP *per capita*, with an APC of 6.74% (95%CI: -1.99 to 16.26; p = 0.51). Among municipalities with higher GDP *per capita*, the APC was 13.91% (95%CI: 4.59 to 24.07; p = 0.06), indicating a borderline trend consistent with a possible increase in rates over the period, although this did not reach statistical significance according to the adopted criterion (Figure 4).

GDP: gross domestic product.

**Figure 5:**
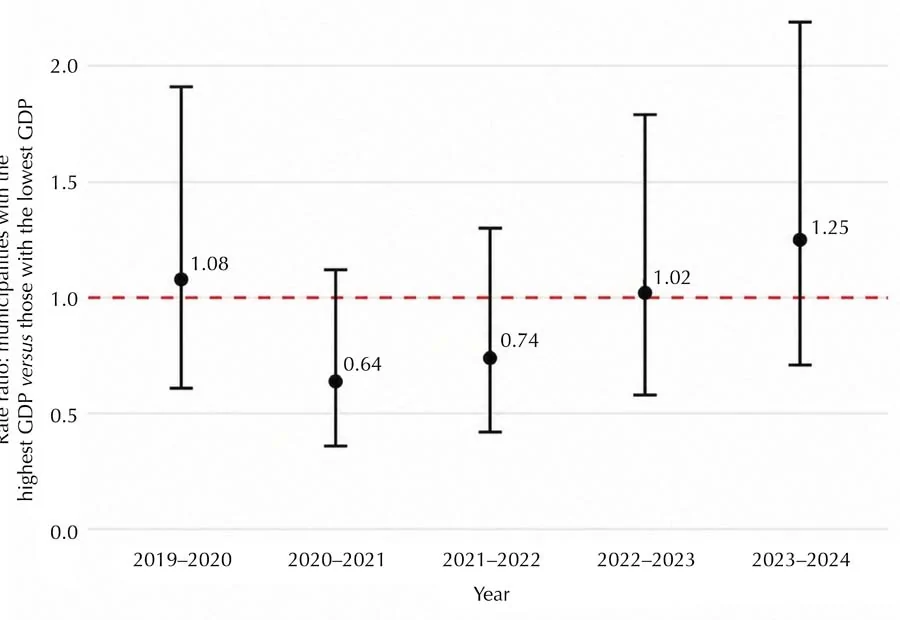
Comparison of the tooth extraction rate between municipalities with the lowest GDP (reference) and those with the highest GDP over time. Brazil, August 2019 to July 2024.

In the multivariate negative binomial mixed-effects model, no significant association was observed between municipal wealth group and tooth extraction rate (β = 0.078; SE = 0.290; p = 0.789) (Figure 5). However, the interactions between group and period were significant in 2020 (β = −0.529; SE = 0.154; p < 0.001) and 2021 (β = −0.377; SE = 0.156; p = 0.016), indicating that, in those years, municipalities with higher GDP *per capita* showed a smaller relative increase in tooth extraction rates compared to municipalities with lower GDP *per capita*. In the within-group comparison, municipalities with lower GDP showed a significant increase in tooth extraction rates in 2021 (β = 0.460; SE = 0.107; p < 0.001), 2022 (β = 0.382; SE = 0.107; p < 0.001), and 2023 (β = 0.349; SE = 0.107; p = 0.001).

Among the variables analyzed in the adjusted model, dental coverage was positively associated with the rate of tooth extractions (β = 0.013; SE = 0.003; p < 0.001). On the other hand, the MHDI showed an inverse association with tooth extraction rates (β = -3.852; SE = 1.663; p = 0.021). The remaining variables did not show a significant association.

## DISCUSSION

### Structural Inequalities and Social Determinants of Tooth Extraction

The results of this study reinforce the relevance of social determinants in the occurrence of tooth extractions in Brazil. Although GDP *per capita* was used as a criterion for stratifying municipal groups, this indicator did not show a significant association with tooth extraction rates in the adjusted model. This finding suggests that municipal economic resources, when analyzed in isolation, have limited ability to explain differences in tooth extraction rates observed in primary health care.

On the other hand, the MHDI showed a significant inverse association with the outcome, indicating that municipalities with better human development conditions tend to have lower tooth extraction rates. This result is consistent with the literature, which demonstrates that municipalities with better social indicators have historically shown a lower burden of oral diseases, greater use of preventive practices, and more timely access to dental services^5,6,21^. Even in light of recent changes in the organization of PHC^9,22^, the MHDI remained an important contextual predictor of oral health, reinforcing the influence of social conditions on health outcomes ^23^.

In addition to human development, population coverage for oral health was also associated with tooth extraction rates. The observed positive association suggests that municipalities with greater availability of dental teams may have a greater capacity to identify, record, and address accumulated dental treatment needs. Thus, the result should not be interpreted as an adverse effect of expanded coverage, but rather as a possible reflection of greater access to and utilization of services in response to existing demand, revealing that organizational factors and the attributes of the PHC teams’ work processes play a central role in the performance of oral health services^24^.

The absence of a significant association between GDP *per capita*, the Gini index, and population density indicates that economic and demographic indicators, taken in isolation, were insufficient to explain the differences observed among the municipalities. Taken together, these findings suggest that aspects related to human development and the organization of oral health services^24^ may play a more significant role in determining tooth extraction rates than municipal wealth itself, potentially mitigating, even if only partially, differences associated with socioeconomic profile^25^.

Finally, the persistence of high tooth extraction rates in both groups highlights the continued prevalence of this procedure in the dental care provided by the SUS. Although the aggregated data does not allow for a comprehensive characterization of the care model’s orientation, the results suggest that differences in socioeconomic context were not accompanied by significant reductions in tooth extraction rates over the analyzed period.

### Temporal Trends, the Pandemic, and Trends in Tooth Extraction Rates

The results of this study show that the number of tooth extractions performed within the SUS remained high over time. The temporal analysis demonstrated that, between August 2019 and July 2024, no significant reduction in the number of tooth extractions was observed, indicating that this procedure continues to play a significant role in the public health system’s response to the population’s oral health needs.

Although the overall trend was classified as stationary according to the statistical criterion adopted, it was observed that municipalities with higher GDP showed a borderline PHC (p = 0.06), consistent with a possible increase in rates over the period. This pattern should be interpreted in the context of the Covid-19 pandemic, a period during which primary care dental services faced restrictions, reorganizations, and changes in the profile of care demand^26^. The reduction in elective appointments, the prioritization of dental emergencies, and the subsequent resumption of services may have contributed to fluctuations in the number of tooth extractions performed. This context includes the fulfillment of pent-up demand in the post-pandemic period and the possible worsening of untreated oral conditions during the period of greatest care restrictions—aspects previously identified as recent impacts on oral health policies^26^. Thus, the increase observed in the final years of the time series should be understood as a possible reflection of cyclical changes in the supply of and demand for dental procedures, and not necessarily as evidence of a permanent trend or a structural failure of the system.

A trend toward convergence of rates between municipalities with higher and lower GDP was observed over the period, with a slight reversal of the initial pattern. Although this trend did not represent a statistically significant difference between the groups in the annual comparisons, it warrants attention from a public health perspective. High socioeconomic indicators may mask intra-urban inequities, since favorable averages do not always reflect equitable access to health services. In this context, it is plausible that, even in municipalities with better socioeconomic indicators, socially vulnerable groups face barriers to access and limitations in the capacity of PHC to identify, monitor, and manage higher-risk cases. Furthermore, although a higher MDHI is associated with better average oral health conditions^22,27^, tooth loss is significantly influenced by individual factors, such as race, income, education level, and history of service use^4,6,28,29^, leading to delayed care and the continued reliance on tooth extraction as a last resort in emergency situations.

On the other hand, the greater stability observed among municipalities with lower GDP may reflect the persistence of accumulated needs and limitations in the healthcare response within socially more vulnerable contexts, which historically have had a lower availability of procedures within the PHC system^2^. Additionally, the predominance of municipalities in the Northeast region in the total proportion of tooth extractions throughout the entire analyzed period reflects, in part, the larger population served by the SUS in that region, associated with historically more unfavorable socioeconomic indicators. The consistent pattern observed across the five macro-regions corroborates previous findings pointing to territorial inequalities in access to and quality of oral health care^9,24^.

In summary, the findings of this study indicate that the persistence of high tooth extraction rates over time, combined with the lack of variation based on municipal GDP and the convergence among groups, signals the existence of persistent challenges for reorienting dental care within PHC, even in more favorable economic contexts.

### Implications for Public Health Policy

Although tooth extractions, taken in isolation, do not comprehensively characterize oral health care, and available records do not allow for the identification of users’ clinical causes or individual trajectories, their analysis at the aggregate level makes it possible to visualize patterns of care provision, municipal trends, and inequalities in PHC. The Ministry of Health also recognizes tooth extractions as a relevant indicator for monitoring oral health, as they reflect the accumulation of oral health problems and their relationship with social and contextual determinants^30^.

The results of this study highlight the importance of public policies that go beyond expanding population coverage and prioritize improving the quality of care, by strengthening longitudinal care, focusing on expanding preventive practices, and improving the problem-solving capacity of PHC^9^. Overcoming regional inequalities also requires financing and organizational strategies capable of guiding the allocation of resources and efforts according to the epidemiological and structural needs of each territory, especially in historically more vulnerable regions. These measures have already been recommended in national evaluations of the Política Nacional de Saúde Bucal (National Oral Health Policy)^26^ and take on even greater relevance considering the stagnant trend observed in tooth extraction rates in the present study.

### Limitations

This study has limitations inherent to the use of secondary data from health information systems, which may contain underreporting, inconsistencies in data entry, and variations in the completeness of the databases. Municipalities with less developed technological infrastructure may face barriers related to connectivity, equipment availability, and staff training, which can affect the consistency of healthcare service records. However, the use of aggregated data over a continuous 60-month time window helps mitigate the impact of one-off fluctuations in reporting. Furthermore, while the five-year time frame does not allow for an assessment of long-term historical trends or the cumulative impacts of the Política Nacional de Saúde Bucal (National Oral Health Policy) since its implementation, it covers a period during which Sisab was already at a more advanced stage of institutional consolidation, thereby reducing the impact of the most intense period of transition among information systems. Furthermore, to minimize potential data losses resulting from the transition between systems, data were obtained simultaneously from SIA/SUS and Sisab, with the aim of enhancing the completeness of the tooth extractions recorded during the analyzed period.

The use of tooth extractions as an aggregate outcome calls for caution in interpreting the results, especially since the study did not incorporate complementary indicators of restorative and preventive procedures. Nevertheless, this is a procedure recorded in a standardized manner in national information systems and recognized as relevant for monitoring oral health at the population level, as it reflects the accumulation of oral health conditions over the course of a lifetime, influenced by social determinants, socioeconomic conditions, and contextual characteristics of the regions^30^. Thus, although tooth extractions do not allow for the characterization of the healthcare model in isolation, they constitute a relevant ecological indicator for analyzing inequalities and temporal patterns in dental care provision within the SUS.

Another limitation relates to the inclusion of the Covid-19 pandemic period, which impacted on the supply of and demand for dental procedures in the SUS due to the reorganization of services and the prioritization of emergencies. However, the multivariate model incorporated relevant contextual variables, such as the MHDI and oral health coverage, in addition to adjusting for the population size of the municipalities, aiming to reduce potential confounders related to socioeconomic, healthcare, and demographic differences among the analyzed groups. Thus, the patterns observed are consistent with the literature and underscore the importance of future research aimed at deepening our understanding of the mechanisms through which better conditions for human development are associated with more preventive, restorative, and equitable oral health care practices within the SUS.

## CONCLUSION

Tooth extraction rates remained stable during the period analyzed, including the post-pandemic period, with no significant association with municipal wealth. The findings suggest that social and healthcare-related factors, such as human development and oral health coverage, help to better understand the distribution of tooth extractions within the SUS. This reinforces the importance of policies aimed at reducing social inequalities, improving the quality of primary care, and strengthening preventive and conservative practices in oral health.

## Footnotes

a Available from: https://ivs.ipea.gov.br/#/

b Avalialble from: https://cidades.ibge.gov.br/

c Available from: http://tabnet.datasus.gov.br

d Available from: https://sisab.saude.gov.br/
