# Supplementary material for: Municipal wealth and tooth extractions in the Unified Health System: limits to changing the healthcare model
Source: Rev Saude Publica. 2026 Sep 2;60:e38. doi: 10.11606/s1518-8787-2026060007600 (PMC13574334; doi:10.11606/s1518-8787-2026060007600)
Supplement: Translation

# Translation: Riqueza municipal e exodontias no Sistema Único de Saúde: limites para mudar o modelo assistencial

**Authors:** Cristiane Maria da Costa Silva, Juliane Franco Martins, Gustavo Fernando Silva, Walbert de Andrade Vieira, Carlos José Soares, Luiz Renato Paranhos

**Affiliations:** I Brasil Universidade Federal de Uberlândia. Faculdade de Odontologia. Programa de Pós-Graduação. Uberlândia, MG, Brasil; II Brasil Universidade Federal de Uberlândia. Faculdade de Odontologia. Uberlândia, MG, Brasil; III Brasil Centro Universitário das Faculdades Associadas de Ensino. Faculdade de Odontologia. São João da Boa Vista, SP, Brasil; IV Brasil Universidade Federal de Uberlândia. Faculdade de Odontologia. Departamento de Dentística e Materiais Dentários. Uberlândia, MG, Brasil; V Brasil Universidade Federal de Uberlândia. Faculdade de Odontologia. Departamento de Ortodontia. Uberlândia, MG, Brasil

**Keywords:** Exodontia, Sistema Único de Saúde, Atenção Primária à Saúde, Série temporal, Indicadores de Desenvolvimento

## Abstract

**OBJETIVO:**

Analisar a tendência temporal das exodontias realizadas na atenção primária à saúde e sua associação com indicadores de desenvolvimento humano e cobertura de saúde bucal em municípios situados nos extremos da distribuição de riqueza municipal.

**MÉTODOS:**

Estudo ecológico com dados secundários de agosto/2019 a julho/2024, tendo municípios brasileiros como unidades de análise. Foram selecionados 200 municípios, correspondentes aos 100 com menor e aos 100 com maior produto interno bruto *per capita*. O número de exodontias foi obtido dos sistemas nacionais de informação em saúde, e as taxas foram calculadas por 1.000 habitantes. A tendência temporal foi estimada por regressão generalizada de mínimos quadrados com estrutura autorregressiva, calculando-se a variação percentual anual. A comparação entre os grupos foi realizada por modelo de efeitos mistos com distribuição binomial negativa, ajustado por indicadores de cobertura de saúde bucal, socioeconômicos e demográficos. Considerou-se nível de significância de 5%.

**RESULTADOS:**

Foram registradas 355.218 exodontias no período. As taxas apresentaram comportamento estatisticamente estacionário nos dois grupos, embora os municípios com maior produto interno bruto *per capita* tenham apresentado comportamento limítrofe de incremento (p = 0,06). O Índice de Desenvolvimento Humano Municipal apresentou associação inversa com o desfecho e cobertura populacional de saúde bucal apresentou associação positiva. O produto interno bruto *per capita* não apresentou associação significativa com as taxas de exodontia no modelo ajustado.

**CONCLUSÃO:**

A manutenção das taxas de exodontia ao longo da série histórica, independentemente da riqueza municipal, sugere que fatores relacionados ao desenvolvimento humano e à organização dos serviços podem ser mais relevantes para compreender a distribuição desse procedimento no Sistema Único de Saúde.

## INTRODUÇÃO

As exodontias figuram entre os procedimentos odontológicos mais realizados no Sistema Único de Saúde (SUS) e constituem indicador relevante do padrão de acesso na Atenção Primária à Saúde (APS)^1,2^. Embora sejam intervenções necessárias, sua elevada frequência pode refletir acesso tardio, baixa resolutividade clínica e persistência de desigualdades sociais no cuidado odontológico^3,4^. No Brasil, essas desigualdades se manifestam de forma heterogênea entre municípios com diferentes níveis de desenvolvimento humano, influenciando tanto a demanda quanto a capacidade de oferta dos serviços^5,6^.

Regiões com menores índices de desenvolvimento humano (IDH) tendem a apresentar piores indicadores de acesso^7^, maior carga de doenças bucais^8^ e de diagnóstico tardio do câncer de boca^9^. Entretanto, a realização de exodontias permanece pouco explorada sob a perspectiva das desigualdades entre os municípios. Entre as causas de perda dental, encontra-se a cárie^10,11^, seguida das doenças periodontais e dos traumas^12,13^, que representam problemas globais de saúde pública, principalmente quando se considera que bilhões de pessoas não têm acesso a cuidados básicos de saúde bucal^14^. Quando o tratamento conservador não é viável, a exodontia torna-se a opção mais acessível financeiramente para o alívio da dor^15,16^. Nesse cenário, a manutenção de elevadas taxas de exodontia pode reforçar desigualdades em saúde bucal.

A perda de elementos dentários pode impactar significativamente a qualidade de vida do indivíduo, pois, além das implicações estéticas que afetam as interações sociais, há também a deterioração funcional que prejudica a fala e a performance mastigatória^16^, ainda que a reabilitação protética seja uma opção. Em contextos de piores indicadores sociais e econômicos, o acesso aos serviços de saúde bucal^17^e, consequentemente, à reabilitação protética^18^ é frequentemente mais restrito, o que potencializa os efeitos sociais e funcionais da perda dentária.

Apesar da relevância do tema, ainda não está suficientemente esclarecido se diferenças na riqueza municipal são capazes de influenciar o padrão temporal de realização de exodontias na APS. Esse conhecimento é fundamental para identificar desigualdades estruturais, orientar estratégias de regionalização e fortalecer políticas de saúde bucal baseadas em evidências. Assim, o objetivo deste estudo foi analisar a tendência temporal das exodontias realizadas na atenção primária do SUS e sua associação com indicadores municipais de riqueza e desenvolvimento humano.

## MÉTODOS

### Tipo de Estudo

Trata-se de um estudo observacional ecológico com recorte temporal, baseado em dados secundários de domínio público. Por não envolver identificação individual, o estudo dispensa apreciação por Comitê de Ética em Pesquisa, estando de acordo com a Resolução do Conselho Nacional de Saúde nº 510/2016. O relato deste estudo seguiu as recomendações do STROBE (*Strengthening the Reporting of Observational Studies in Epidemiology*)^19^ e de sua extensão RECORD (*REporting of studies Conducted using Observational Routinely-collected health Data*)^20^, específica para pesquisas baseadas em dados secundários rotineiramente coletados.

### Participantes

A população do estudo foi composta por municípios brasileiros, considerados como unidades de análise, classificados segundo o produto interno bruto (PIB) *per capita* municipal. Foram incluídos todos os municípios que apresentaram registro válido de exodontias no SUS no período de agosto de 2019 a julho de 2024, conforme dados dos sistemas de informações do Ministério da Saúde.

Para fins analíticos, os municípios foram estratificados em dois grupos extremos de riqueza municipal, definidos a partir da distribuição do PIB *per capita*: um grupo composto pelos 100 municípios com menor PIB *per capita* e outro pelos 100 municípios com maior PIB *per capita*. Essa seleção teve caráter analítico e exploratório, com o objetivo de comparar contextos econômicos marcadamente distintos e verificar se diferenças acentuadas de riqueza municipal estariam associadas às taxas de exodontia na atenção primária. Foram incluídos no estudo todos os municípios pertencentes a um desses dois estratos que apresentaram ao menos um registro de exodontia no período avaliado. Municípios com ausência total de registros do procedimento foram excluídos das análises.

### Dimensionamento e Seleção da Amostra

Realizou-se dimensionamento da amostra com o objetivo de assegurar número suficiente de municípios para comparação entre os estratos socioeconômicos. Considerou-se como referência para o cálculo amostral, o número de municípios classificados como de alta vulnerabilidade social pelo Instituto de Pesquisa Econômica Aplicada (IPEA)^a^, intervalo de confiança de 95% e erro amostral de 10%, acrescido de 5% de margem de segurança para compensar possíveis inconsistências nos dados.

A partir desses parâmetros, definiu-se a inclusão de 100 municípios em cada grupo, correspondendo aos extremos da distribuição do PIB *per capita* municipal. Para a seleção, todos os municípios brasileiros foram organizados em ordem crescente de PIB *per capita*, conforme dados do Instituto Brasileiro de Geografia e Estatística (IBGE)^b^, sendo selecionados os 100 municípios com menor e os 100 com maior PIB *per capita*.

### Variáveis

A variável dependente foi o número de exodontias de dentes decíduos e permanentes realizadas no período analisado, identificadas pelos códigos 04.14.02.012-0 (decíduos) e 04.14.02.013-8 (permanentes).

As variáveis independentes incluíram: PIB *per capita*, Índice de Desenvolvimento Humano Municipal (IDHM), Índice de Gini, cobertura populacional de saúde bucal da atenção primária, população total e densidade demográfica dos municípios selecionados.

### Coleta de Dados

Os dados referentes aos procedimentos de exodontias realizados pelos municípios brasileiros foram obtidos junto ao Departamento de Informática do Sistema Único de Saúde (Datasus), por meio de dois sistemas: Sistema de Informações Ambulatoriais (SIA/SUS)^c^ e Sistema de Informação em Saúde para a Atenção Básica (Sisab)^d^, ambos de acesso público.

A utilização concomitante desses sistemas justifica-se pelas mudanças ocorridas na organização do registro da produção ambulatorial da APS no Brasil ao longo do período analisado, especialmente após a consolidação do e-SUS atenção primária. Assim, a integração das informações foi necessária para assegurar completude e continuidade temporal dos dados, minimizando perdas de informação decorrentes da transição entre sistemas.

A identificação dos procedimentos de exodontia foi realizada a partir dos códigos oficiais do Sistema de Gerenciamento da Tabela de Procedimentos, Medicamentos e OPM do SUS (SIGTAP), sendo incluídos exclusivamente os registros correspondentes aos procedimentos de exodontia de dentes permanentes (04.14.02.013-8) e decíduos (04.14.02.012-0), realizados entre agosto de 2019 e julho de 2024. Procedimentos de exodontia de dentes supranumerários foram excluídos.

**Tabela:** Características sociodemográficas e taxas de extração dentária dos municípios incluídos no estudo, de acordo com o grupo de riqueza municipal.

	Maior PIB m (DP)	Menor PIB m (DP)
PIB *per capita*	128.321,9 (147,4)	7.180,7 (150,7)
População	39.030,06 (74.539,0)	16.214,78 (8.633,5)
Cobertura populacional de saúde bucal da atenção primária	76,5 (32,4)	91,7 (18,4)
Índice de Desenvolvimento Humano	0,699 (0,06)	0,557 (0,03)
Índice de Gini	0,40 (0,04)	0,37 (0,03)
Densidade populacional	168,96 (560,79)	26,74 (24,79)
Taxa de exodontia^a^	11,2 (14,7)	19,1 (18,4)
Média total de exodontias	271,9 (771,7)	288,3 (303,6)

PIB: Produto Interno Bruto; DP: desvio padrão.

^a^ Taxa por 1.000 habitantes.

PIB: Produto Interno Bruto.

A cobertura populacional de saúde bucal da atenção primária foi obtida do sistema e-Gestor, do Ministério da Saúde. As informações socioeconômicas municipais foram obtidas por meio do IBGE, incluindo população total e densidade demográfica (Censo 2022) e PIB *per capita* (2021). O IDHM foi obtido a partir do portal do IBGE e o Índice de Gini por meio do Datasus. Os dados foram tabulados em planilhas do Microsoft Excel e organizados por ano, região, estado e município.

### Tratamento dos Dados

O tratamento dos dados incluiu a verificação de duplicidades, identificadas por registros com mesmas chaves de informação (município, competência, código do procedimento e quantidade) nos sistemas de informações, mantendo-se apenas um registro válido por ocorrência. Foi realizada ainda uma análise de consistência temporal e numérica, com exclusão de valores manifestamente incompatíveis com padrões históricos de produção municipal, reduzindo potenciais vieses decorrentes de erros de registro. Após essas etapas, os dados foram agregados por município e por ano, constituindo a base analítica final.

### Análise dos Dados

Para avaliar a tendência temporal das taxas de exodontias ao longo do período de cinco anos, foram calculadas taxas ajustadas por 1.000 habitantes para cada município. A tendência anual foi estimada separadamente para os grupos de municípios de menor e maior PIB *per capita* por meio de regressão generalizada de mínimos quadrados com estrutura autorregressiva de primeira ordem [GLS AR (1)]. A partir do coeficiente da variável tempo, foi calculada a variação percentual anual (APC), considerando p > 0,05 como tendência estacionária, valores positivos como aumento e negativos como redução.

Para comparar as taxas de exodontias entre os grupos ao longo do período, foi ajustado um modelo de efeitos mistos generalizado (GLMM) com distribuição binomial negativa (nbinom2). O desfecho foi o número de procedimentos, ajustado pelo logaritmo da população municipal como offset (por 1.000 habitantes). Foram incluídos efeitos fixos para grupo socioeconômico, ano, IDHM, Índice de Gini e densidade populacional, cobertura populacional de saúde bucal da atenção primária, bem como a interação entre grupo e ano. O município foi incluído como efeito aleatório, a fim de corrigir a correlação intramunicipal. Os resultados foram apresentados como razão de taxas (*rate ratio* – RR), com intervalos de confiança de 95% e valores de p. As análises foram realizadas no *software* R (versão 4.5.1).

## RESULTADOS

As análises incluíram 200 municípios brasileiros (100 com menor e 100 com maior PIB *per capita*), selecionados conforme os critérios previamente descritos. No período agosto de 2019 a julho de 2024, os municípios selecionados realizaram 355.218 exodontias no âmbito do SUS. Desse total, 51,8% foram executadas por municípios classificados no grupo de menor PIB. A Tabelasintetiza as características dos municípios analisados segundo os grupos de riqueza municipal. De modo geral, os municípios de menor PIB *per capita* apresentaram maior cobertura de saúde bucal e maior taxa média de exodontias por 1.000 habitantes, enquanto os municípios de maior PIB *per capita* concentraram maiores valores médios de densidade populacional e IDHM.

A Figura 1 apresenta a evolução temporal do número absoluto de exodontias realizadas nos municípios incluídos no estudo, segundo os grupos de riqueza municipal, bem como o total agregado de procedimentos. No primeiro período avaliado (agosto de 2019 a julho de 2020), os municípios de maior PIB *per capita* apresentaram maior número absoluto de exodontias em comparação aos municípios de menor PIB *per capita*. Entre agosto de 2020 e julho de 2022, observou-se maior número absoluto de procedimentos nos municípios de menor PIB *per capita*. Nos dois períodos subsequentes, os valores tornaram-se mais semelhantes entre os grupos. Considerando o conjunto dos municípios analisados, o número total de exodontias aumentou entre agosto de 2020 e julho de 2023, seguido de discreta redução no período de agosto de 2023 a julho de 2024, mantendo-se, entretanto, acima dos valores observados no início da série histórica.

**Figura 1:** Número de procedimentos de exodontia realizados na Atenção Primária à Saúde em municípios nos extremos de PIB *per capita*. Brasil, agosto de 2019 a julho de 2024.

PIB: Produto Interno Bruto.

Nota: dados extraídos de forma integrada do Sistema de Informações Ambulatoriais (SIA/SUS) e do Sistema de Informação em Saúde para a Atenção Básica (SISAB) do Ministério da Saúde. O “Total dos dois grupos” representa o somatório dos procedimentos realizados nos 200 municípios analisados.

A Figura 2 apresenta a distribuição anual das taxas de exodontia por 1.000 habitantes segundo o grupo de riqueza municipal. Em todos os períodos avaliados, os municípios de menor PIB *per capita* apresentaram medianas superiores às dos municípios de maior PIB *per capita*. Apesar disso, observou-se ampla variabilidade intragrupo ao longo de toda a série, com maior dispersão em alguns períodos e ocorrência de valores extremos em ambos os estratos.

**Figura 2:** Taxa anual de exodontias no SUS em municípios brasileiros, de acordo com o PIB municipal. Brasil, agosto de 2019 a julho de 2024.

Nota: dados integrados do Sistema de Informações Ambulatoriais (SIA/SUS) e do Sistema de Informação em Saúde para a Atenção Básica (SISAB) do Ministério da Saúde. Os valores representam a participação percentual de cada macrorregião no total de procedimentos executados pelos municípios da amostra.

A Figura 3 apresenta a distribuição proporcional das exodontias segundo macrorregião ao longo do período analisado. Em todos os anos, a maior proporção de procedimentos concentrou-se entre os municípios da região Nordeste, seguida pelas regiões Sudeste e Norte. As regiões Sul e Centro-Oeste apresentaram as menores participações relativas. Esse padrão se manteve relativamente estável ao longo da série, com variações discretas entre os períodos avaliados.

**Figura 3:** Proporção anual de exodontias realizadas pelas macrorregiões brasileiras no SUS. Brasil, agosto de 2019 a julho de 2024.

PIB: Produto Interno Bruto.

Na análise de tendência temporal, as taxas de exodontia por 1.000 habitantes apresentaram comportamento estacionário nos municípios de menor PIB *per capita*, com APC de 6,74% (IC95% -1,99 a 16,26; p = 0,51). Entre os municípios de maior PIB *per capita*, observou-se APC de 13,91% (IC95% 4,59 a 24,07; p = 0,06), indicando comportamento limítrofe e compatível com possível incremento das taxas ao longo do período, embora sem atingir significância estatística segundo o critério adotado (Figura 4).

**Figura 4:** Variação percentual anual da taxa de extrações dentárias por 1.000 habitantes nos municípios com maior PIB e com menor PIB. Brasil, agosto de 2019 a julho de 2024.

PIB: Produto Interno Bruto.

No modelo multivariado de efeitos mistos binomial negativo, não foi observada associação significativa entre grupo de riqueza municipal e taxa de exodontia (β = 0,078; SE = 0,290; p = 0,789) (Figura 5). Entretanto, as interações entre grupo e período foram significativas em 2020 (β = -0,529; SE = 0,154; p < 0,001) e 2021 (β = -0,377; SE = 0,156; p = 0,016), indicando que, nesses anos, os municípios de maior PIB *per capita* apresentaram menor aumento relativo das taxas de exodontia em comparação aos municípios de menor PIB *per capita*. Na comparação intragrupo, os municípios com menor PIB apresentaram aumento significativo nas taxas de extração em 2021 (β = 0,460; SE = 0,107; p < 0,001), 2022 (β = 0,382; SE = 0,107; p < 0,001) e 2023 (β = 0,349; SE = 0,107; p = 0,001).

**Figura 5:** Comparação da taxa de extração dentária entre os municípios com menor PIB (referência) e com maior PIB ao longo do tempo. Brasil, agosto de 2019 a julho de 2024.

Entre as variáveis analisadas no modelo ajustado, a cobertura de saúde bucal esteve positivamente associada à taxa de extrações dentárias (β = 0,013; SE = 0,003; p < 0,001). Por outro lado, o IDHM apresentou associação inversa com as taxas de exodontia (β = -3,852; SE = 1,663; p = 0,021). As demais variáveis não apresentaram associação significativa.

## DISCUSSÃO

### Desigualdades Estruturais e Determinantes Sociais da Exodontia

Os resultados deste estudo reforçam a relevância dos determinantes sociais na ocorrência de exodontias no Brasil. Embora o PIB *per capita* tenha sido utilizado como critério de estratificação dos grupos municipais, esse indicador não apresentou associação significativa com as taxas de exodontia no modelo ajustado. Esse achado sugere que a disponibilidade econômica municipal, quando analisada isoladamente, possui capacidade limitada para explicar diferenças nas taxas de extração dentária observadas na APS.

Por outro lado, o IDHM apresentou associação significativa e inversa com o desfecho, indicando que municípios com melhores condições de desenvolvimento humano tendem a registrar menores taxas de exodontia. Esse resultado é consistente com a literatura, que demonstra que municípios com melhores indicadores sociais apresentam, historicamente, menor carga de doenças bucais, maior uso de práticas preventivas e acesso mais oportuno aos serviços odontológicos^5,6,21^. Mesmo diante das mudanças recentes na organização da APS^9,22^, o IDHM manteve-se como um importante preditor contextual da saúde bucal, reforçando a influência das condições sociais sobre os desfechos em saúde^23^.

Além do desenvolvimento humano, a cobertura populacional de saúde bucal também se associou às taxas de exodontia. A associação positiva observada sugere que municípios com maior disponibilidade de equipes podem apresentar maior capacidade de identificar, registrar e atender necessidades acumuladas de tratamento odontológico. Dessa forma, o resultado não deve ser interpretado como efeito adverso da ampliação da cobertura, mas como possível expressão de maior acesso e utilização dos serviços frente à demanda existente, revelando que fatores organizacionais e os atributos do processo de trabalho das equipes de APS exercem papel central no desempenho dos serviços de saúde bucal^24^.

A ausência de associação significativa entre PIB *per capita*, índice de Gini e densidade populacional indica que indicadores econômicos e demográficos, isoladamente, não foram suficientes para explicar as diferenças observadas entre os municípios. Em conjunto, esses achados sugerem que aspectos relacionados ao desenvolvimento humano e à organização dos serviços de saúde bucal^24^ podem exercer papel mais relevante na determinação das taxas de exodontia que a riqueza municipal propriamente dita, podendo atenuar, mesmo que parcialmente, diferenças associadas ao perfil socioeconômico^25^.

Por fim, a manutenção das taxas de exodontia em ambos os grupos evidencia a persistência desse procedimento no cuidado odontológico ofertado pelo SUS. Embora os dados agregados não permitam caracterizar de forma abrangente a orientação do modelo assistencial, os resultados sugerem que as diferenças de contexto socioeconômico não foram acompanhadas por reduções expressivas das taxas de exodontia ao longo do período analisado.

### Tendência Temporal, Pandemia e Comportamento das Taxas de Exodontia

Os resultados deste estudo evidenciam as taxas de exodontia não apresentaram redução significativa ao longo do período analisado. A análise temporal demonstrou que, entre agosto de 2019 e julho de 2024, não se observou redução significativa na produção de extrações dentárias, indicando que esse procedimento continua desempenhando papel relevante na resposta dos serviços públicos às necessidades de saúde bucal da população.

Embora a tendência global tenha sido classificada como estacionária segundo o critério estatístico adotado, observou-se que municípios de maior PIB apresentaram APC limítrofe (p = 0,06), compatível com possível incremento das taxas ao longo do período. Esse comportamento deve ser interpretado à luz do contexto da pandemia de covid-19, período em que os serviços odontológicos da atenção primária sofreram restrições, reorganizações e mudanças no perfil da demanda assistencial^26^. A redução de atendimentos eletivos, a priorização de urgências odontológicas e posterior retomada dos serviços podem ter contribuído para oscilações na produção de exodontias. Esse contexto inclui o atendimento de demanda reprimida no período pós-pandêmico e o possível agravamento de condições bucais não tratadas durante o período de maior restrição assistencial, aspectos previamente apontados como impactos recentes nas políticas de saúde bucal^26^. Assim, o aumento observado nos anos finais da série deve ser compreendido como possível reflexo de mudanças conjunturais na oferta e na demanda por procedimentos odontológicos, e não necessariamente como evidência de tendência permanente ou de falha estrutural do sistema.

Observou-se tendência de aproximação das taxas entre municípios com maior e menor PIB ao longo do período, com discreta inversão do padrão inicial. Embora esse movimento não tenha representado diferença estatisticamente significativa entre os grupos nas comparações anuais, ele merece atenção do ponto de vista da saúde pública. Indicadores socioeconômicos elevados podem ocultar iniquidades intraurbanas, uma vez que médias favoráveis nem sempre traduzem acesso equitativo aos serviços de saúde. Nesse contexto, é plausível que, mesmo em municípios com melhores indicadores socioeconômicos, grupos socialmente vulneráveis enfrentem barreiras de acesso e limitações na capacidade da APS em identificar, acompanhar e manejar casos de maior risco. Ademais, embora o maior IDHM esteja associado a melhores condições médias de saúde bucal^22,27^, a perda dentária sofre influência marcante de fatores individuais, como raça, renda, escolaridade e histórico de uso dos serviços^4,6,28,29^, favorecendo trajetórias assistenciais tardias e a manutenção da exodontia como resposta final frente a situações de urgência.

Por outro lado, a estabilidade observada entre municípios de menor PIB pode refletir a persistência de necessidades acumuladas e limites na resposta assistencial, em contextos socialmente mais vulneráveis, que historicamente apresentam menor oferta de procedimentos no âmbito da APS^2^. Adicionalmente, a predominância de municípios da região Nordeste na proporção total de exodontias ao longo de todo o período analisado pode estar relacionada, entre outros fatores, à composição regional da amostra e às desigualdades socioeconômicas historicamente observadas. O comportamento consistente observado nas cinco macrorregiões corrobora achados prévios que apontam desigualdades territoriais no acesso e na qualidade da atenção em saúde bucal^9,24^.

Em síntese, os achados deste estudo indicam que manutenção das taxas de exodontia ao longo do tempo, associada à ausência de diferenciação segundo o PIB municipal e à aproximação entre os grupos, sinaliza a existência de desafios persistentes para a reorientação do cuidado odontológico na APS, mesmo em contextos econômicos mais favoráveis.

### Implicações para a Política de Saúde Pública

Embora a exodontia, isoladamente, não qualifique de forma abrangente a assistência em saúde bucal e registros disponíveis não permitam identificar a causa clínica ou trajetória individual dos usuários, sua análise em nível agregado permite visualizar padrões de produção assistencial, tendências municipais e desigualdades na APS. O Ministério da Saúde também reconhece as exodontias como indicador relevante para monitoramento da saúde bucal, por refletirem o acúmulo de agravos bucais e sua relação com determinantes sociais e contextuais^30^.

Os resultados deste estudo apontam para a importância de políticas públicas que ultrapassem a ampliação da cobertura populacional e priorizem a qualificação da atenção, com o fortalecimento da longitudinalidade, foco na ampliação de práticas preventivas e melhora da capacidade resolutiva da APS^9^. A superação das desigualdades regionais também requer estratégias de financiamento e organização capazes de orientar a alocação de recursos e esforços conforme as necessidades epidemiológicas e estruturais de cada território, especialmente nas regiões historicamente mais vulneráveis. Essas medidas já vêm sendo indicadas em avaliações nacionais da Política Nacional de Saúde Bucal^26^ e ganham ainda maior relevância diante do comportamento estacionário observado das taxas de extração dentária no presente estudo.

### Limitações

Este estudo apresenta limitações inerentes ao uso de dados secundários provenientes dos sistemas de informação em saúde, os quais podem conter sub-registros, heterogeneidades no preenchimento e variações na completude das bases. Os municípios com menor infraestrutura tecnológica podem enfrentar barreiras relacionadas à conectividade, disponibilidade de equipamentos e treinamento de pessoal, o que pode influenciar a regularidade dos registros da produção assistencial. Contudo, a utilização de dados agregados ao longo da janela temporal contínua de 60 meses contribui para reduzir o peso de oscilações pontuais de notificação. Além disso, o recorte temporal de cinco anos, embora não permita avaliar tendências históricas de longo prazo nem os impactos acumulados da Política Nacional de Saúde Bucal desde sua implantação, refere-se a um período em que o Sisab já se encontrava em estágio mais avançado de consolidação institucional, reduzindo o impacto do período mais intenso de transição dos sistemas de informação. Adicionalmente, para reduzir possíveis perdas decorrentes da transição entre sistemas, os dados foram obtidos simultaneamente do SIA/SUS e do Sisab, buscando ampliar a completude da produção de exodontias captada no período analisado.

A utilização das exodontias como desfecho agregado impõe cautela à interpretação dos resultados, especialmente porque o estudo não incorporou indicadores complementares de procedimentos restauradores e preventivos. Ainda assim, trata-se de procedimento registrado de forma padronizada nos sistemas nacionais de informação e reconhecido como relevante para o monitoramento da saúde bucal em nível populacional, pois reflete o acúmulo de agravos bucais ao longo da vida, influenciado por determinantes sociais, condições socioeconômicas e características contextuais dos territórios^30^. Dessa forma, embora as exodontias não permitam caracterizar isoladamente o modelo assistencial, constituem indicador ecológico pertinente para analisar desigualdades e padrões temporais da produção odontológica no SUS.

Outra limitação refere-se à inclusão do período da pandemia de covid-19, que impactou a oferta e a demanda por procedimentos odontológicos no SUS devido à reorganização dos serviços e à priorização de urgências. Porém, o modelo multivariado incorporou variáveis contextuais relevantes, como IDHM e cobertura de saúde bucal, além de ajuste para o porte populacional dos municípios, buscando reduzir potenciais confundimentos relacionados às diferenças socioeconômicas, assistenciais e demográficas entre os grupos analisados. Assim, os padrões observados mostram-se consistentes com a literatura e reforçam a importância de investigações futuras que aprofundem a compreensão dos mecanismos pelos quais melhores condições de desenvolvimento humano se associam a práticas de cuidado em saúde bucal mais preventivas, restauradoras e equitativas no âmbito do SUS.

## CONCLUSÃO

As taxas de exodontia mantiveram-se estáveis no período analisado, inclusive no período pós-pandêmico, sem associação significativa com a riqueza municipal. Os achados sugerem que dimensões sociais e assistenciais, como desenvolvimento humano e cobertura de saúde bucal, ajudam a compreender melhor a distribuição de exodontias no SUS. Isso reforça a importância de políticas voltadas à redução das desigualdades sociais, à qualificação da atenção primária e ao fortalecimento de práticas preventivas e conservadoras em saúde bucal.

## Footnotes

a Disponível em: https://ivs.ipea.gov.br/#/

b Disponível em: https://cidades.ibge.gov.br/

c Disponível em: http://tabnet.datasus.gov.br

d Disponível em: https://sisab.saude.gov.br/
